# Complete chloroplast genome sequence of turnip (*Brassica rapa*. ssp. *rapa*): genome structure and phylogenetic analysis

**DOI:** 10.1080/23802359.2020.1829124

**Published:** 2020-10-09

**Authors:** Rui Han, Minyu Tian, Guangnan Zhang, Dengkui Shao, Yanjing Ren

**Affiliations:** a Academy of Agriculture and Forestry Sciences of Qinghai University (Qinghai Academy of Agriculture and Forestry Sciences), Xining, P. R. China; b Qinghai Key Laboratory of Vegetable Genetics and Physiology, Xining, P. R. China

**Keywords:** Turnip, chloroplast genome, genome structure, phylogenetic analysis

## Abstract

Turnip (*Brassica rapa.* ssp. *rapa*) is considered worldwide to be one of the most important leaf and root vegetable crops in the Brassicaceae family. However, to date, few chloroplast (cp) genomic resources have been reported for this genus. Here, we determined the complete cp genome sequences of *Brassica rapa* ssp. *rapa.* A 153,621 bp quadripartite cycle without any gap was obtained with a large single-copy region (LSC) of 83,512 bp, a small single-copy region (SSC) of 17,683 bp, and two inverted repeat (IR), IRa and IRb of 26,213 bp. A total of 132 genes were identified, including 87 protein-coding genes (PCG), 37 transfer RNA (tRNA), and 8 ribosomal RNA (rRNA). The phylogenetic analysis of ten other crops selected showed that the turnip was most closely related to the *Brassica rapa*.

Turnip (*Brassica rapa* ssp. *rapa* L. 2*n* = 2× =20), one of the most important leaf and root vegetable crops in the Brassicaceae family, is widely spread planted in China and throughout East Asia. As a vegetable crop, turnip is rich in glucosinolates (Rochfort et al. 2006; Zhang et al. 2008), dietary phenolic (Chung et al. 2016), dietary fiber, vitamin C and other bioactive compounds (Thiruvengadam and Chung 2015). The complete chloroplast (cp) genome of angiosperms usually comprises four parts: a large single-copy region (LSC), a small single-copy region (SSC) and two inverted repeats (IR), IRa and IRb that reside between LSC and SSC, finally forming a typical quadripartite cycle (Li et al. 2017). Compared with the nuclear genome, the cp genome is inherited from the maternal parent, which is highly conserved in gene content and genome structure. Usually, the length of cp genomes ranges from 120 to 160 kb and the number of unique genes range from 110 to 130 (Du et al. 2020). Genes in the cp genome play crucial roles in photosynthesis and the biosynthesis of starch, amino acids, fatty acids, and pigments (Rodríguez-Ezpeleta et al. 2005).

Traditionally, turnip is considered to be closest to the *Brassica rapa* even if its phenotype is similar to the *Raphanus sativus*. In recent years, the cp genomes of many species in the Brassicaceae family were reported, and several phylogenetic analysis were performed to show the phylogenetic relationships among Brassicaceae species (Prabhudas et al. 2015; Seol et al. 2015; Du et al. 2020). However, to our knowledge, this is the first complete turnip cp genome sequence presented. The phylogenetic relationships were performed between seven Brassicaceae species, three other species (*Oryza sativa*, *Solanum lycopersicum* and *Nicotiana tabacum*) and turnip. These results of this research will provide better understanding on evolutionary relationships among Brassicaceae species and turnip.

The fresh and healthy leaf of *B. rapa* ssp. *rapa* was collected from an individual turnip plant, W21, which planted in the field of Qinghai university (N36°42′; E 101°45′), Xining, China, and its genomic DNA was stored at Qinghai university. The DNA extraction followed by the modified CTAB (cetyl trimethyl ammonium bromide) method (Porebski et al. 1997). DNA purification, library construction and assessment were performed as detailed in the manufacturer’s protocol. The qualified DNA were sequenced using an Illumina HiSeq2500 with an average read length of 1363 bp (Shaanxi Breeding Biotechnologies Co., Ltd). After raw reads were filtered to obtain high quality data by removing the adaptor reads and reads of low quality, a total of 8.38 Gb clean data with a Q30 value of 91.65% was obtained. The draft cp genome of turnip was assembled using the MITObim software (Hahn et al. 2013) with *Brassica nigra* (NC_030450) as a reference. The draft genome was further corrected by PE read mapping. Genes in the complete cp were annotated by the DOGMA (Wyman et al. 2004) and Mitofy (Alver Son et al. 2010) software.

The complete cp genome of turnip (GenBank accession no. MT409177) displayed a quadripartite cycle of 153,621 bp without any gaps, and consisting of four regions: a large single-copy region (LSC) of 83,512 bp, a small single-copy region (SSC) of 17,683 bp, and two inverted repeat (IR), IRa and IRb, of 26,213bp. In the turnip cp genome, 132 distinctive genes were identified, including 87 protein-coding genes, 37 tRNAs, and 8 rRNA. Of these 132 genes, there were 15 genes with two copies in the IR regions. They were *ndhB*, *rrn4.5*, *rrn5*, *rrn16*, *rrn23*, *rps7*, *rps12*, *rpl2*, *rpl23*, *trnI-CAU*, *trnV-GAC*, *trnN-GUU*, *ycf1*, *ycf2, and ycf15*, respectively.

The base composition of the complete cp genome sequence was analyzed and the overall GC content was 36.3%. The content of G and C was 18.6 and 17.8%, respectively. The overall GC content in IR regions (42.3%) were higher than that in the LSC (34.1%) and SSC regions (29.3%). The distribution of GC content in turnip cp genome was similar with that in *Lagerstroemia* (Zheng et al. 2020). Conversely, the overall AT content in SSC region (71.9%) was higher than that in the IR region (57.7%), and the content of A and T were 31.4 and 32.3%, respectively.

Phylogenetic relationships between the ten species and turnip were conducted by MEGA7 using the maximum likelihood (ML) method and 1000 bootstrap replicates with the Oryza sativa (NC 031333.1) as an outgroup. The results showed that *B. rapa* ssp. *rapa* (MT409177) was closely related to *B. rapa* (NC040849.1), while *B. nigra* (KT878383.1) is more diverse than the neighboring species *Raphanus sativus* (NC022469.1) (Figure 1). These results were consistent with other studies (Jeong et al. 2014; Seol et al. 2015), and supports that turnip is a sub-species of *B. rapa*.

**Figure 1.**
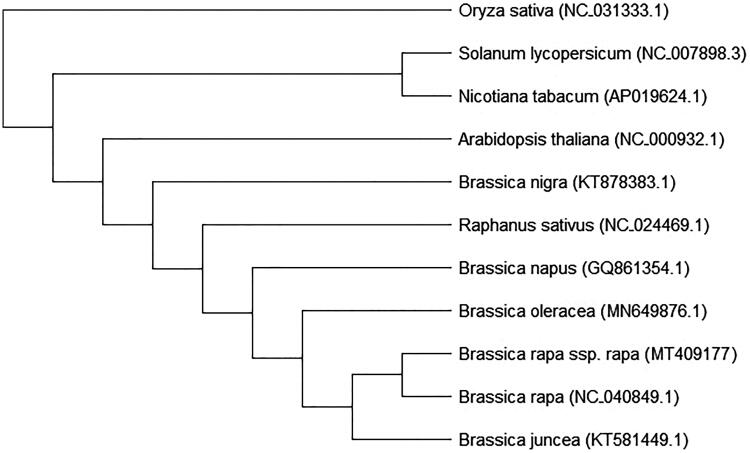
Phylogenetic analysis of 11 species based on the chloroplast protein-coding sequences. The chloroplast sequence of Oryza sativa (NC 031333.1) was used as the outgroup.

## Data Availability

The data that support the findings of this study are openly available in NCBI at https://www.ncbi.nlm.nih.gov/nuccore/MT409177 with the accession no. MT409177.
